# Infectious RNA vaccine protects mice against chikungunya virus infection

**DOI:** 10.1038/s41598-020-78009-7

**Published:** 2020-12-03

**Authors:** Inga Szurgot, Karl Ljungberg, Beate M. Kümmerer, Peter Liljeström

**Affiliations:** 1 Department of Microbiology, Tumor and Cell Biology, Karolinska Institutet, 171 77 Stockholm, Sweden; 2 Institute of Virology, Medical Faculty, University of Bonn, Bonn, Germany; 3 Present Address: Eurocine Vaccines AB, Karolinska Institutet Science Park, 171 65 Solna, Sweden

**Keywords:** RNA vaccines, Alphaviruses

## Abstract

We describe a novel vaccine platform that can generate protective immunity to chikungunya virus (CHIKV) in C57BL/6J mice after a single immunization by employing an infectious RNA (iRNA), which upon introduction into a host cell launches an infectious attenuated virus. We and others have previously reported that an engineered deletion of 183 nucleotides in the nsP3 gene attenuates chikungunya virus (CHIKV) and reduces in vivo viral replication and viremia after challenge in mice, macaques and man. Here, we demonstrated that in vitro transfection of iRNA carrying the nsP3 deletion generated infectious viruses, and after intramuscular injection, the iRNA induced robust antibody responses in mice. The iRNA was superior at eliciting binding and neutralizing antibody responses as compared to a DNA vaccine encoding the same RNA (iDNA) or a non-propagating RNA replicon (RREP) lacking the capsid encoding gene. Subsequent challenge with a high dose of CHIKV demonstrated that the antibody responses induced by this vaccine candidate protected animals from viremia. The iRNA approach constitutes a novel vaccine platform with the potential to impact the spread of CHIKV. Moreover, we believe that this approach is likely applicable also to other positive-strand viruses.

## Introduction

Chikungunya virus (CHIKV) is mainly spread by two mosquito vectors, *Aedes aegypti* and *Aedes albopictus*. *Aedes aegypti* is endemic in the tropics whereas *Aedes albopictus* is invasive and has established populations also in temperate climate zones. *Aedes aegypti* was responsible for all recorded CHIKV epidemics prior to the new millennium, when an adaptation enabled efficient transmission by *Aedes albopictus* mosquitoes. This caused CHIKV to re-merge in 2004 and caused a large outbreak in La Réunion. Since then, large epidemics driven by transmission through both vectors have been recorded in Africa and Asia, and has caused local transmission in Europe^1,2^. More recently, CHIKV has infected millions of people across the Americas. Human symptoms include acute febrile illness, debilitating polyarthritis, which can be prolonged for years and, in rare cases, severe encephalitis. Currently, there is no licensed vaccine to prevent the disease caused by CHIKV infection. Early attempts using an attenuated CHIKV isolated from a clinical sample as a vaccine were discontinued because of severe side effects, probably due to reversion of the attenuation^3,4^, and it was later determined that the attenuation was due to only a few point-mutations^5^. However, live attenuated vaccines constitute some of the most potent counter-measures to infectious diseases to date. Using a reverse genetics approach, we have described two CHIKV containing large engineered deletions corresponding to 60 amino acids in their genomes that lead to attenuations that are incredibly unlikely to reverse but yet allow viral replication in cell culture and permissive animal models^6,7^. The deletions have been located in the nsP3 and 6K genes, respectively. The nsP3 protein has important functions for the alphavirus replicase, and deletions in its C terminal hypervariable parts affect interactions with several host proteins^8–12^. Importantly, immunization with the Δ5nsP3 virus was able to confer protective immune responses both in mice, and non-human primates (NHP)^6,7^. In the latter case, the protective responses lasted for at least a year after a single immunization^7^. Recently, this approach has also been shown to be safe and very immunogenic after a single immunization in clinical trials^13^.

We and others have previously explored a plasmid DNA vaccine approach in which attenuated viruses capable of inducing protective immunity in mice are launched from a cytomegalovirus (CMV) promoter, sometimes referred to as infectious DNA or iDNA^6,14–18^. Using a cDNA-launched vaccine should eliminate the accumulation of mutations that can occur during the growth of vaccine stocks of in particular RNA viruses.

Clinical grade DNA vaccines are produced by large scale bacterial fermentation. It might, however, be desirable to have a vaccine entity that could be produced synthetically in a cell free system. Recent advances in RNA production technology facilitate production of large quantities of GMP grade RNA. Moreover, the development of RNA nucleoside modifications has decreased innate immune activation while increasing in vivo RNA stability, translational activity and immunogenicity^19–21^. RNA may also offer other potential advantages over plasmid DNA as a vaccine modality. For instance, DNA vaccines need to be delivered to the cell nucleus to become transcriptionally active, whereas an RNA only requires cytosolic delivery. Two major types of RNA vaccines have been utilized against infectious pathogens: self-replicating replicon RNA (RREP) vaccines and non-replicating mRNA vaccines^22^. Most currently used self-replicating RREP vaccines are based on an alphavirus genome^23^, where the genes encoding the RNA replication machinery are intact but the genes encoding the structural proteins are replaced with the antigen of interest. The RREP platform enables a large amount of antigen production from a single RNA copy owing to intracellular replication and amplification of the antigen-encoding RNA^24^.

We and others have previously described that non-propagating self-replicating alphavirus replicon RNA vaccines provide protection against a number of infectious diseases in small animal models^25–30^. Here we describe a novel strategy to address the threat of CHIKV as an emerging infectious disease by using an in vitro transcribed RNA coding for an attenuated variant of CHIKV. By analogy to iDNA, we will refer to this approach as infectious RNA or iRNA. The difference between iDNA and iRNA is that transcription of iDNA requires entry into the cell nucleus, a process that is believed only to occur in dividing cells^31^. In contrast, the positive-strand iRNA is active in the cytoplasm and will initiate the replicative cycle upon entry into any cell. In the cytoplasm, the 5′ proximal portion of the genome encoding the viral replicase genes nsP1-4 is immediately translated to produce the viral replicase. The replicase then replicates the incoming iRNA and subsequently transcribes mRNA encoding the viral capsid and glycoproteins from a subgenomic viral promoter. This results in the assembly of new virions which are released by budding from the plasma membrane. Thus, the iRNA approach combines the strength of reverse genetics and the potency of live attenuated vaccines.

In the present study, we have engineered an iRNA construct, and compared it to the previously described iDNA and RREP platforms. Initial studies in vitro showed that transfection of iRNA into BHK-21 cells generated infectious viruses, and subsequent evaluation in a mouse infection model demonstrated that the iRNA vaccine candidate was highly immunogenic after a single low-dose immunization and was able to confer protection against challenge with a high dose of CHIKV. We therefore believe that the iRNA platform described here represents a promising vaccine candidate against CHIKV worth further investigation for clinical development.

## Method

### CHIKV and vaccine candidates

Construction of the wildtype infectious clone CHIKV LR2006-OPY1, referred to here as CHIKV, has been described^32,33^. Plasmids for production of infectious RNA (iRNA) were generated by cloning cDNAs of CHIKV mutants Δ5nsP3 under the control of an SP6 promoter as described^6^. Template plasmid for production of RNA replicon (RREP) encoding CHIKV envelope protein was generated by cloning the cDNAs of CHIKV devoid of the capsid-encoding sequence (residues 7565–8350 of the CHIKV genome) under the control of an SP6 RNA polymerase promoter as described^34^. The resulting DNA templates were subsequently linearized with NotI, and subsequently iRNA and RREP were produced by in vitro transcription using mMessage mMachine SP6 kit (Life Technologies, Carlsbad, CA). Kit reactions include cap analog [m7G(5′)ppp(5′)G] incorporated as the first or 5′ terminal G of the transcript. The DNA template was subsequently removed from the reaction mixture by DNAse treatment and RNA was purified using RNAeasy purification kit (Qiagen, Valencia, CA). A plasmid vector capable of producing infectious viruses (iDNA) was previously constructed by cloning the cDNA of ΔnsP3 strain under the control of the human CMV immediate-early promoter^6^. All resulting clones were verified by sequencing.

### Mice and immunizations

The vaccine candidates were tested in 8-week-old female C57BL/6J mice (Charles River, Germany). Animals were kept at the Astrid Fagraeus laboratory, Karolinska Institutet, in accordance with the recommendations of the Swedish Board of Agriculture. The protocol was approved by the local ethics committee, Stockholms norra djurförsöksetiska nämnd, permit number N82/14, and all animal procedures were performed according to the approved guidelines and regulations. iRNA, iDNA or RREP preparations were diluted in 50 μl of nuclease-free water (Life Technologies, Carlsbad, CA) and inoculated intramuscularly in the gastrocnemius muscle of the left hind leg, at the doses of 0.125, 1.25 or 10 μg, as indicated in the figure legends.

### CHIKV ELISA

Detection of antibodies was done essentially as described (5). For calculation of endpoints titers, a cutoff value based on the OD values from the naive controls was determined (2 times average from naïve control sera at the lowest dilution + 2 standard deviations, typically at an OD = 0.1). The endpoint titers for the samples with the OD490 redouts below the cut-off value were set to 10 for plotting and subsequent statistical analyses. Titers were determined by interpolating the point where the sigmoid curve reaches the cutoff value using the GraphPad Prism 7 software.

### CHIKV neutralization assay

Neutralizing antibody titers were determined in all available pre-challenge serum samples from mice immunized with iRNA Δ5nsP3 (n = 10), iDNA Δ5nsP3 (n = 10) or naïve controls (n = 5) mainly as described previously using chikungunya virus replicon particles (VRPs) expressing Gaussia luciferase (Gluc)^35^. Briefly, BHK-J cells (kindly provided by Charles M. Rice, New York) were seeded in 96-well plates at 2 × 10^4^ cells per well. The next day, VRPs (MOI 5) were preincubated with twofold serial dilutions of heat inactivated serum samples for 1 h at 37 °C before the mixture was added to the 96-well plates. After 1 h at 37 °C, the inoculum was removed and cells were washed once with PBS before media was added. Readout of secreted Gluc was performed at 24 h after infection. Neutralization potency was determined as a percentage of determined Gluc activity compared to the Gluc readout after VRP infection without serum. Results are presented as 50% neutralization (NT_50_) titers.

### Mouse challenge model

Five weeks after immunizations, C57BL/6J mice were infected subcutaneously (s.c.) at the dorsal side of each hind foot with a total of 10^6^ plaque forming units (pfu) CHIKV diluted in 2 × 20 µl phosphate-buffered saline (PBS) as described previously^6,36^.

### Viremia

Titers of virus particles in serum collected at day 1–7 after immunization with 1.25 µg of iRNA constructs, and at day 2 post challenge with wildtype virus corresponding to the viremia peak were determined by plaque assay. Blood samples were collected in Microtainer tubes (BD) at four different time points between day 1 and day 7, and serum was obtained by centrifugation for 90 s at 5000 rpm in a microcentrifuge. Serum was diluted in minimum essential medium (MEM) containing 0.2% bovine serum albumin (BSA) (Gibco/Life Technologies) and tested by plaque assay as described previously^31^. Briefly, confluent BHK-21 cells in 12-well plates were washed with PBS twice, serum or supernatants harvested from iRNA transfected BHK-21 cells in serial dilutions were added to the wells, and the wells were incubated at 37 °C in 5% CO_2_ for 1 h. The infection medium was removed, and 1 ml of overlay medium, consisting of 1 part of 2% carboxymethyl cellulose (CMC, Avicel, Belgium) in PBS and 2 parts of complete BHK-21 medium (BHK-21 Glasgow MEM supplemented with tryptose phosphate broth, 0.1 U/ml penicillin, 0.1 μg/ml streptomycin 2 mM l-glutamine, 10 mM HEPES, and 10% fetal bovine serum [Gibco/Life Technologies]), was added to each well. Plates were incubated at 37 °C in 5% CO_2_. After 48 h, 1 ml of 10% formaldehyde–PBS was added to the cells and the reaction mixture was incubated at room temperature for at least 4 h. Wells were subsequently washed with PBS, and crystal violet solution (0.1% crystal violet in 20% methanol) was added until clear plaques appeared. Wells were rinsed in tap water and the plaques counted. Viremia data are presented as the peak viremia of each mouse.

### Statistics

Statistical analysis was performed using GraphPad Prism 7 software (GraphPad Software Inc., La Jolla, CA, USA). We used the nonparametric Mann–Whitney U test for pairwise analysis between two groups and the Kruskal Wallis test followed by Dunn’s test for group wise comparisons, (*p* < 0.05 used to indicate significance).

## Results

### Constructs and in vitro production of propagating virus

**Figure 1:**
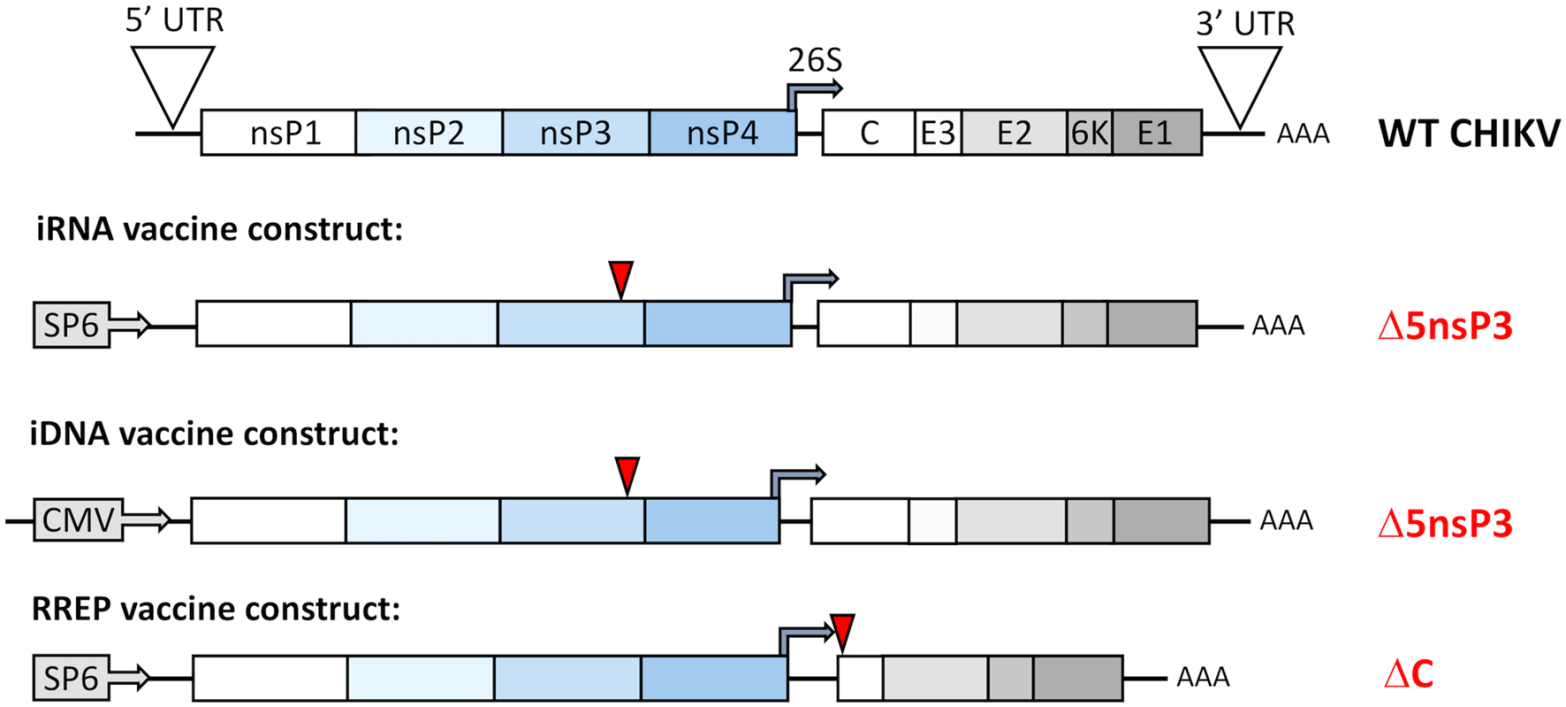
CHIKV vaccine candidates. Schematic representation of CHIKV genome and the vaccine candidates; iRNA Δ5nsP3 has a 183-bp deletion in the 3′ part of the sequence encoding nsP3. For comparison, we used an iDNA Δ5nsP3 mutant launched as DNA infectious genome and the RNA replicon (RREP). The RREP vaccine candidate is derived from the WT CHIKV infectious clone after deletion of the capsid encoding region which prevents RNA packaging and virion assembly. Deletions are indicated by arrows.

**Figure 2:**
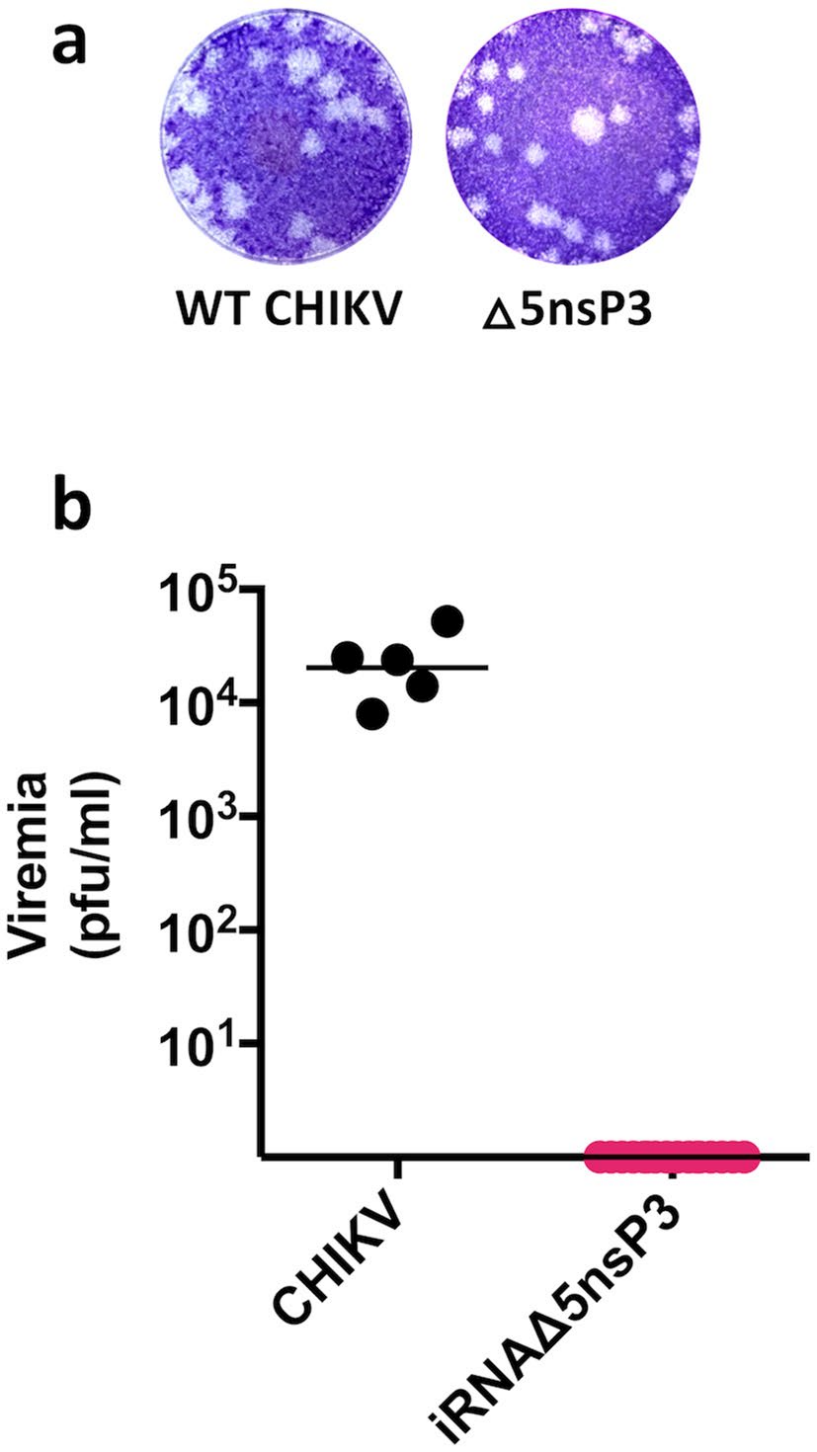
In vitro and in vivo propagation of iRNA vaccine candidate. (**a**) Plaque assay of CHIKV and virus made from the iRNA encoding the Δ5nsP3 deletion. iRNA was electroporated into BHK-21 cells, supernatants were harvested after 48 h and used to infect a monolayer of BHK-21 cells that was analyzed for plaque formation 48 h post infection. (**b**) Peak viremia in serum after a single intramuscular injection of 1.25 μg of iRNA vaccine (n = 16) or subcutaneous injection of 10^6^ pfu of WT CHIKV (n = 5). The limit of detection for the plaque assay was 80 pfu/ml. The line indicates the geometric mean of each group.

The iRNA (Fig. 1) construct was generated using standard molecular cloning techniques. The iRNA encode the CHIKV genome under an SP6 promoter, and contain an attenuating deletion of 183 nucleotides in the nsP3 gene (iRNA Δ5nsP3). We have also prepared constructs containing deletions of the entire 180 nucleotide 6K gene (iRNA Δ6K) or a combination of both deletions (iRNA Δ5nsP3 Δ6K) (Suplementary data, Figures S1, S2). However, in our iRNA experiments, we observed that the iRNA Δ5nsP3 was the most immunogenic vaccine candidate in mice and induced higher antibody titers than the iRNA Δ6K or the iRNA Δ5nsP3Δ6K with a double deletion (see Supplementary Figure S3). Therefore, we decided to proceed with the iRNA Δ5nsP3. For comparisons, we included an iDNA Δ5nsP3 where the SP6 promoter is replaced with a cytomegalovirus promoter, and an RNA replicon that does not encode the CHIKV capsid and thus does not launch propagating viruses. Previously, we have demonstrated that transfection with plasmid-encoded iDNA constructs encoding the Δ5nsP3 deletion generated replicating viruses in cell culture but did not induce viremia in mice^6^. However, we have demonstrated that the Δ5nsP3 virus replicon particles induce viremia in NHPs, which is a more permissive animal model for CHIKV infection^7^, and it was recently shown that vaccination with Δ5nsP3 virus replicon particles also causes viremia in human volunteers^13^. Here, we show that in vitro transcribed iRNA electroporated into BHK-21cells promoted production of viruses able to replicate in cell culture as demonstrated by plaque assay (Fig. 2a).

After intramuscular immunization with 1.25 μg of the iRNA Δ5nsP3 construct, we sampled blood at four separate time points between day 1 and day 7 post inoculation. At no time point could we detect any viable virus in serum of the inoculated mice (Fig. 2b). This is consistent with the lack of viremia after inoculation with iDNA Δ5nsP3 construct^6^.

### Immune responses

**Figure 3:**
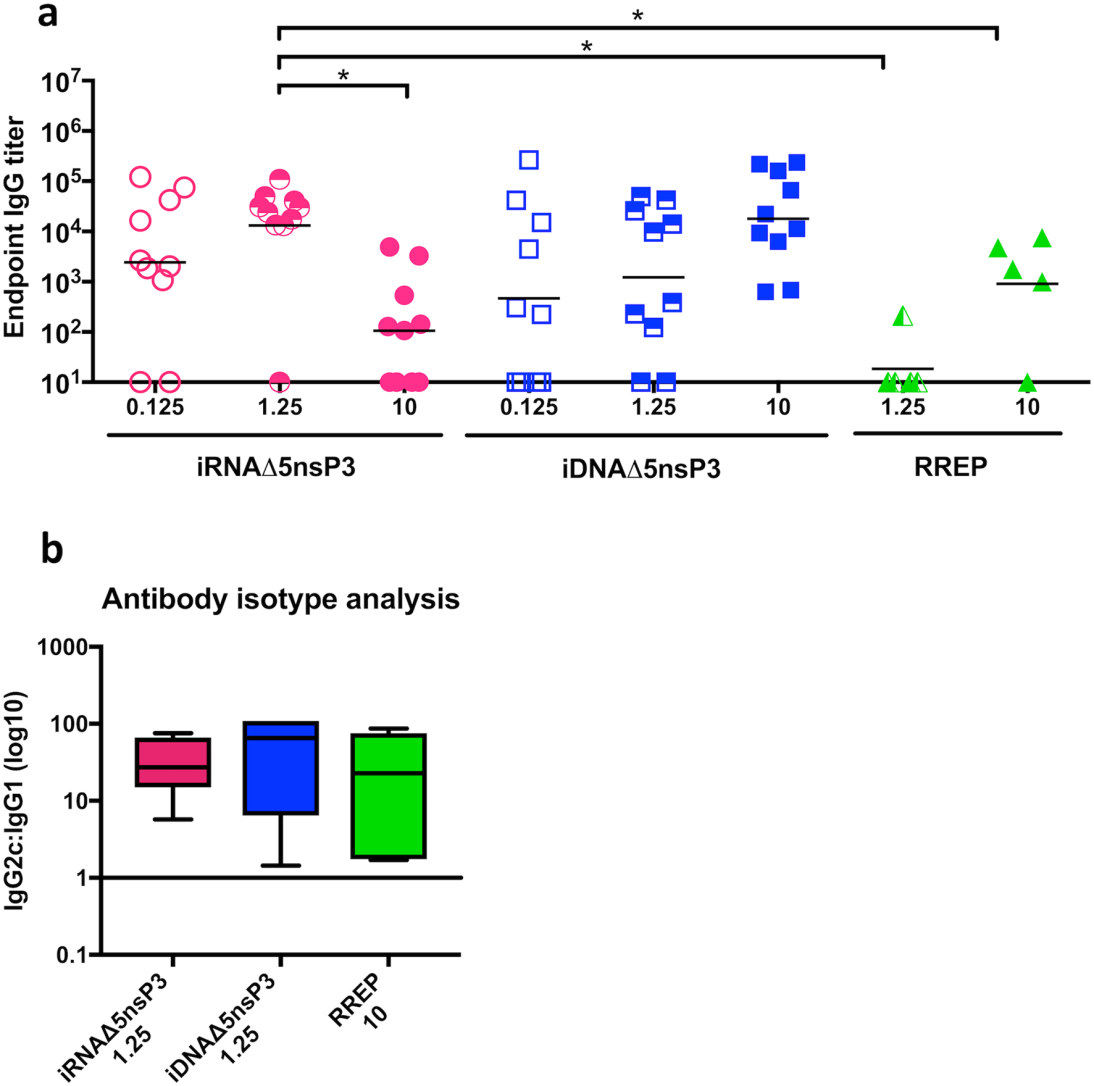
(**a**) Immunogenicity of vaccine candidates. C57BL/6J mice were immunized once with indicated doses of candidate vaccines. Total antigen-specific IgG titers were determined by ELISA three weeks post immunization. The line indicates the geometric mean of each group (n = 10 animals per group). (**b**) Antibody isotype analysis. Sera from immunized were analyzed by ELISA for anti-CHIKV Env p62-E1 protein antibody isotypes IgG2c and IgG1. Results are presented as IgG2c:IgG1 endpoint antibody titer ratios against CHIKV Env p62-E1 protein. A ratio above 1 indicates a Th1 biased response. Non-responders in the binding IgG ELISA were not included in the analysis. For the statistical analysis, we performed Kruskal–Wallis test followed by Dunn’s test for multiple comparisons. One asterisk (*) indicates statistical differences of *p* < 0.05.

In CHIKV infection, antibody responses in general and neutralizing antibody responses in particular constitute the correlate of protection. Thus, serum samples from mice immunized with 0.125, 1.25 or 10 μg unformulated naked iRNA or iDNA, or 1.25 or 10 μg unformulated naked RREP were screened for anti-CHIKV envelope antibodies by ELISA three weeks post immunization (Fig. 3a). Increased doses elicited higher antibody titers in mice immunized with both iDNA and RREP. Interstingly, the iRNA Δ5nsP3 construct elicited higher antibody responses at 0.125 and 1.25 μg doses (geometric mean titer, GMT, of 2416 and 13,096, respectively) than at the 10 μg dose (GMT of 105). The 1.25 μg dose elicited significantly higher endpoint anti-CHIKV titers compared to the 10 μg dose (*p* < 0.05). Moreover, at the 1.25 μg dose, immunization with the iRNA Δ5nsP3 elicited significantly higher antibody responses than immunization with RREP (GMT of 24), (*p* < 0.05). The iRNA-induced antibody GMT was over tenfold higher than the GMT elicited with 1.25 μg dose of the iDNA Δ5nsP3 candidate vaccine (geometric mean of 13,096 vs 1229). Although, in the multiple comparisons analysis used for the multiple groups in this binding antibody screen experiment, the difference was not statistically significant. This observation suggests that the use of iRNA Δ5nsP3 could allow vaccine dose sparing compared to the corresponding iDNA vaccine.

The production of IgG2c and IgG1 are associated with secretion of cytokines consistent with a Th1 or a Th2 profile, respectively, and can be used as an indication of a predominantly Th1 or Th2 biased response. To evaluate how the different vaccine platforms affected Th1 and Th2 induction, we determined the ratio of IgG2c and IgG1 antibody titers against CHIKV spike antigen (Fig. 3b). We observed that mice immunized with all tested constructs induced higher IgG2c antibody titers compared to IgG1, indicating a Th1 biased response. However, it should be noted that the C57BL/6 mice used in this study are genetically predisposed to produce predominantly Th1 helper cells^37^. IgG2c/IgG1 ratios were higher in animals immunized with the iRNA vaccine candidate in comparison to the two other groups, although the difference was not statistically significant.

### Protection from challenge

**Figure 4:**
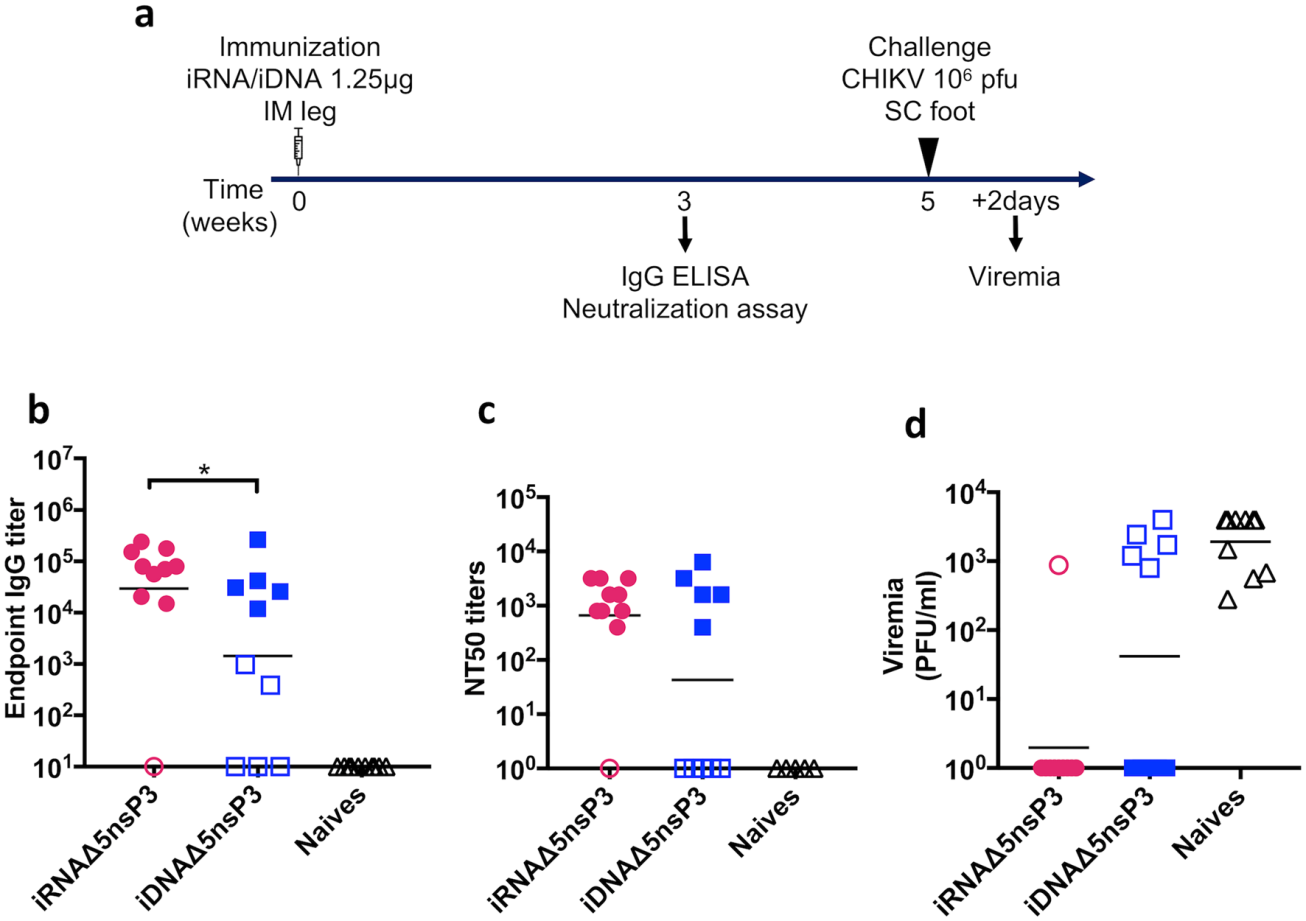
Challenge study. (**a**) Schematic representation of the experiment schedule. C57BL/6J mice were immunized once with 1.25 μg of either iRNA Δ5nsP3 or iDNA Δ5nsP3 and challenged five weeks later with 10^6^ plaque forming units (pfu) of CHIKV. (**b**) Anti-CHIKV IgG endpoint titers and (**c**) 50% neutralization titers (NT_50_) were determined three weeks after a single immunization with the candidate vaccines. (**d**) Viremia was determined by plaque assay using serum samples collected at day two post challenge. Filled symbols indicate mice that were protected against challenge, whereas open symbols indicate mice that were not protected against infection with CHIKV. The line indicates the geometric mean of each group (n = 10 animals per group). A two-tailed Mann–Whitney test was used to analyze differences between two groups. One asterisk (*) indicates *p* < 0.05.

Mice that were immunized with 1.25 μg of either iRNA Δ5nsP3 or iDNA Δ5nsP3 were challenged with 10^6^ plaque forming units (pfu) of CHIKV 5 weeks after a single immunization (Fig. 4a). Two weeks before the time of challenge, the anti-CHIKV antibody responses were determined by ELISA (Fig. 4b) and neutralization assay (Fig. 4c). Consistent with our previous results, we could observe that iRNA Δ5nsP3 elicited higher anti-CHIKV binding antibody responses (*p* > 0.05, Mann–Whitney test) as compared to iDNA Δ5nsP3 immunization.

Although binding antibodies may be indicative of protective immunity, neutralizing antibodies are considered crucial for protection against CHIKV infection. We have previously shown a good correlation between induction of binding and neutralizing antibodies using live attenuated and iDNA Δ5nsP3 vaccine candidates in mice and NHPs^6,7^. To determine whether the antibody responses elicited by iRNA Δ5nsP3 immunization were neutralizing, we used a CHIKV replicon particles (VRPs) neutralization assay^35^. High levels of neutralization antibodies, with NT_50_ values ranging from 400–3200, were detected in the serum samples of 9 out 10 mice in the group immunized with 1.25 μg of Δ5nsP3 iRNA (geometric mean of 666) (Fig. 4c). Immunization with the same dose of iDNA construct elicited the neutralizing antibodies in 5 out of 10 animals with geometric mean NT50 titers of 42. The neutralization titers mirrored the IgG titers for both groups and correlated strongly with protection from challenge (Fig. 4b–d).

In the iRNA immunized mice, nine out of ten mice were protected whereas only five out of ten were protected in the iDNA group (*p* > 0.05, Mann–Whitney test) (Fig. 4c). These data are in accordance with our previous finding that an ELISA titer ≥ 10,000 constitutes a protective level in this challenge model^6,38^. Accordingly, all mice with ELISA titers above 10,000 also developed neutralizing antibody responses.

## Discussion

The most potent antiviral vaccines are based on attenuated strains. Live attenuated vaccines (LAV) have the capacity to elicit both humoral and cellular immune responses capable of preventing infection as well as clearing infected cells and preventing further dissemination. LAV against yellow fever, measles, mumps and rubella are highly effective and elicit long-lasting immunity, sometimes after a single vaccination. With increased understanding of the pathobiology of viruses, the implementation of reverse genetics can be used to engineer attenuations for the efficient development of safe and potent attenuated vaccines^6,39,40^. By launching the attenuated virus from a nucleic acid rather than an infectious virus, a universal platform technology enabling production of several different vaccines using the same manufacturing process can be employed.

We and others have described alphavirus vaccines based on the delivery of a DNA-launched virus or iDNA^6,16,17^. The same strategy has also been employed to generate flavivirus vaccines^14,18,41,42^. Although this approach holds promise, it would be appealing to be able to produce the vaccine without cellular or animal components. We explored the potential of using in vitro transcribed infectious RNA encoding an attenuated CHIKV, and demonstrated the ability of iRNA containing an attenuating deletion, Δ5nsP3, to elicit an anti-CHIKV immune response. This attenuating deletion has been engineered to be large enough to make reversion to wildtype virus incredibly unlikely, and indeed, serial passaging of viruses carrying these deletions did not reveal any revertants^6^. Moreover, vaccination with the attenuated virus has proved safe and immunogenic in NHP and man^7,13^. Naturally, adverse events is a concern for any live attenuated virus vaccine, and the clinical studies revealed a reactogenicity profile that was considered safe in the high-dose group and well tolerated in the low- and medium-dose groups for this vaccine candidate, which further underlines the clinical potential for an iRNA-based candidate based on this attenuation.

The Δ5nsP3 attenuating deletion is targeting a hyper-phosphorylated region of the highly variable domain in the C terminal end of the nsP3 protein that serves as a hub for interactions with cellular factors such as PI3K-Akt-mTOR^11^. It has been shown that deletions in the corresponding region of the closely related SFV nsP3 result in reduced rate of synthesis of viral RNA, and as a consequence, this virus has reduced pathogenicity in mice^12^. Nevertheless, the Δ5nsP3 CHIKV grows to high titers in cell culture and relatively high titers in mice and NHPs^6,7^. It was also a significantly better immunogen than RREP, an RNA encoding the wildtype nsP3 but devoid of the capsid and thus incapable of producing progeny viral particles. The RREP should consequently have a more efficient cytoplasmic RNA replication than the iRNA Δ5nsP3. This arguably means that local propagation of virus particles actually occurs and has an effect on immunogenicity, although we were not able to detect any viral particles in serum samples collected at various time points post iRNA Δ5nsP3 inoculation.

At low doses, iRNA Δ5nsP3 appeared more immunogenic than iDNA Δ5nsP3 despite the absence of any delivery vehicles or formulations that would improve RNA delivery or half-life in the tissue. A tentative explanation for this observation would be that the iRNA is active as an mRNA immediately in the cytoplasm of any transfected cell, whereas an iDNA has to cross both the plasma membrane and the nuclear membrane during cell division in order for RNA transcription to be initiated. It is plausible that immunization with an iRNA therefore has many more potential targets for transfection and thus has a better prospective for decreased dosage than DNA based platforms. Improved delivery thus has a tremendous potential to further improve the iRNA vaccine modality. In addition, RNA is an immunogenically active molecule that can activate innate immune responses and could have an adjuvant effect per se. Moreover, since the infectious dose of CHIKV infection is as low as 10–100 viruses in NHPs^7,43^, the iRNA platform may lead to even more significant vaccine dose sparing compared to other DNA and RNA vaccine platforms. In theory in vivo transfection of only a limited number of cells would be sufficient to launch the attenuated virus. In fact, in the clinical trial with the Δ5nsP3 LAV, a dose of only 3.2 × 10^3^ 50% tissue culture infection dose was immunogenic and elicited seroconversion of 100% of the volunteers^13^ further supports the possibility of using low iRNA doses. However, for the iRNA Δ5nsP3 we see a reverse dose dependence at the highest dose given. There might be several explanations for this. Firstly, alphavirus replicon induction of type I interferon (IFN) production is dose dependent. Given the antiviral activity of type I IFN that rapidly shuts down replication of viral RNA genomes, we hypothesized that lower doses would be equally or more immunogenic than higher doses. Using DNA launched replicons, we have previously established that the type I IFN response has a negative impact on the immune response at increasing vaccine doses. A vigorous type I IFN response leads to quenching of viral expression through induction of an antiviral state. However, we have shown that this negative effect is absent in mice deficient in the type I interferon signaling system^44^. Here, we observe a difference between iRNA and iDNA vaccines, where the dose response curve for iRNA peaks at a lower dose than for iDNA or DREP. One possible explanation for this is that RNA is a more potent inducer of type I interferon than DNA, and that an antiviral state is induced before the viral replication is established. Indeed, a plethora of pattern recognition receptors such as TLR3, TLR7, TLR8, PKR, OAS, RIG-I and MDA5 are designated to recognize viral RNA and transmit danger signals to the immune system through type I IFN signaling^45–49^. In further support of our conclusion, a recent study by Vogel et al., obtained similar results after immunization with various doses of self-replicating replicon RNA^50^.

The fact that submicrogram doses of iRNA vaccines are active after intramuscular delivery without any formulation or enhanced delivery such as electroporation is very promising for further development. We ascribe this feature to the launching of replicating particles in vivo that establish a local, contained infection, although we could not detect systemic viremia in mice. For vaccine safety, this is an important aspect to investigate further in future studies. The lack of viremia is consistent with previous findings that inoculation with iDNA encoding wildtype, Δ5nsP3 or Δ6K viruses do not cause viremia in mice^6^. However, a fraction (40%) of susceptible mice infected with a high titer of Δ5nsP3 virus displayed viremic infection. In these studies, we used a mouse model for CHIKV infection. However, this model is relatively poorly permissive for CHIKV, and viremia typically reaches 10^3^–10^4^ pfu/ml serum after challenge with the wildtype virus. In cynomolgus macaques, viremia induced by inoculation of wildtype CHIKV typically reaches 10^9^ genome copies/ml (which is estimated to correspond to 10^7^ pfu/ml)^7,43^ and in humans 10^6^ pfu/ml^51^. Inoculation of Δ5nsP3 CHIKV in cynomolgus macaques causes a delayed and blunted viremia reaching 10^5^ pfu/ml^7^. Thus, it is possible that iRNA dose sparing in more permissive animal models and future human studies may be even greater.

Due to improved production, manufacturing and storage, RNA based vaccines have recently gained a lot of attention. It has been shown that mRNA vaccines, if protected from RNases can preserve potency after storage at temperatures ranging from – 80 to + 70 °C for several months^52^. In addition, improved in vivo delivery may be instrumental for the success of RNA based vaccines. We have previously demonstrated that intradermal delivery followed by electroporation can increase the immunogenicity of RNA vaccines. Nevertheless, in our studies comparing RREP to mRNA vaccines expressing an identical antigen, the mRNA failed to elicit detectable immune responses^27^. In the study presented here, one immunization with naked unformulated iRNA at a low dose on a per weight basis was needed to elicit protective responses. In comparison, in studies with unmodified, modified or formulated mRNA or self-replicating RREP vaccines, typically two doses of at least ten-fold higher doses have been required^20,21,53–55^.

In conclusion, this brief report demonstrates the potential of iRNA encoding an attenuated CHIKV to generate protective immune responses at very low doses and warrants further investigation of this platform for clinical development.

## Supplementary information

Supplementary Figures.

## Data Availability

The datasets generated during the current study are available from the corresponding author on reasonable request.
