# Supplementary material for: Infectious RNA vaccine protects mice against chikungunya virus infection
Source: Sci Rep. 2020 Dec 3;10:21076. doi: 10.1038/s41598-020-78009-7 (PMC7712826; doi:10.1038/s41598-020-78009-7)
Supplement: Supplementary file 1 — Supplementary Figures. [file 41598_2020_78009_MOESM1_ESM.pdf]

Infectious RNA vaccine protects mice against chikungunya virus infection

Inga Szurgot[1#*], Karl Ljungberg[1,2,#], Beate M. Kümmerer[3], Peter Liljeström[1]

[1] Department of Microbiology, Tumor and Cell Biology, Karolinska Institutet, 171 77 Stockholm, Sweden

[2] Current address: Eurocine Vaccines AB, Karolinska Institutet Science Park, 171 65 Solna, Sweden.

[3] Institute of Virology, Medical Faculty, University of Bonn, Germany

[#] Contributed equally to the work

*** Correspondence:**
Inga Szurgot
inga.szurgot@ki.se

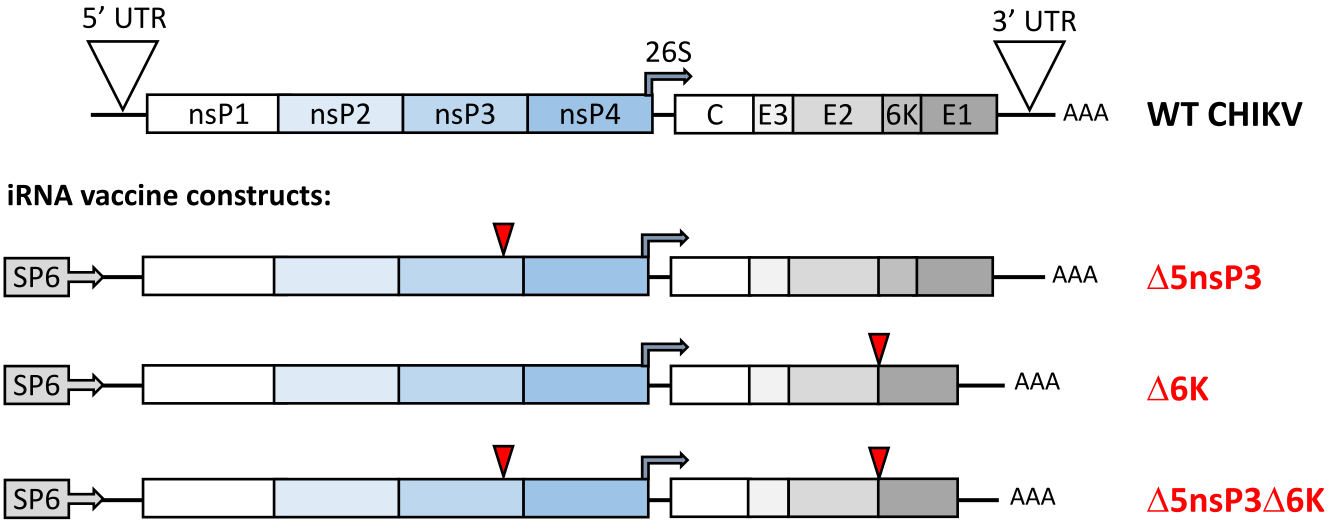

Supplementary Figure 1. iRNA vaccine candidates. Schematic representation of CHIKV genome and the three iRNA vaccine candidates; iRNA Δ5nsP3, iRNA Δ6K and the iRNA Δ5nsP3Δ6K. Δ5nsP3 has a 183-bp deletion in the 3' part of the sequence encoding nsP3, and Δ6K is devoid of 6K. Deletions are indicated by arrows.

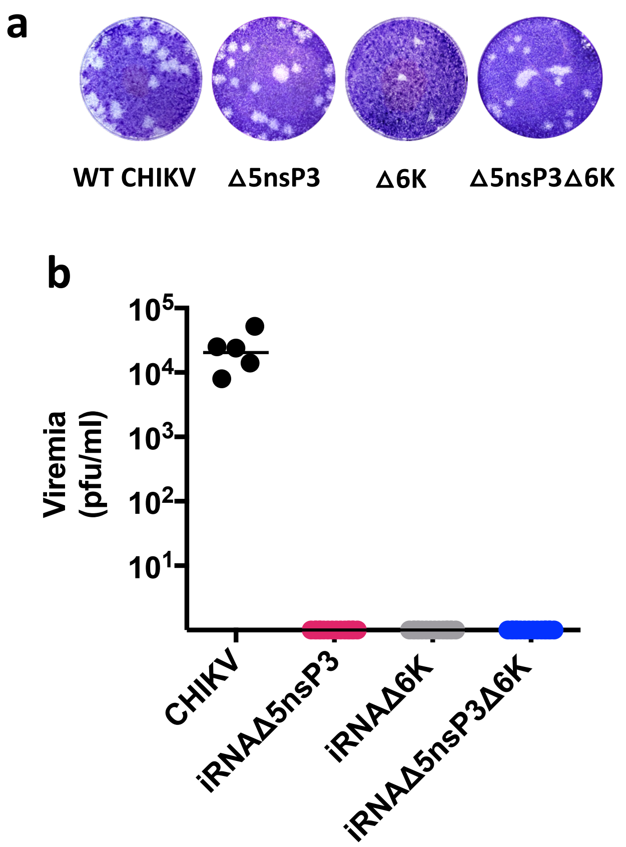

Supplementary Figure 2. *In vitro* and *in vivo* propagation of vaccine candidates. (a) Plaque assay of CHIKV and virus made from the iRNA encoding the Δ5nsP3, Δ6K and Δ5nsP3Δ6K deletions. iRNA was electroporated into BHK-21 cells, supernatants were harvested after 48h and used to infect a monolayer of BHK-21 cells that was analyzed for plaque formation 48h post infection. (b) Peak viremia in serum after a single intramuscular injection of 1.25µg iRNA vaccines (n=16) or subcutaneous injection of 10^6 pfu of WT CHIKV (n=5). The line indicates the geometric mean of each group.

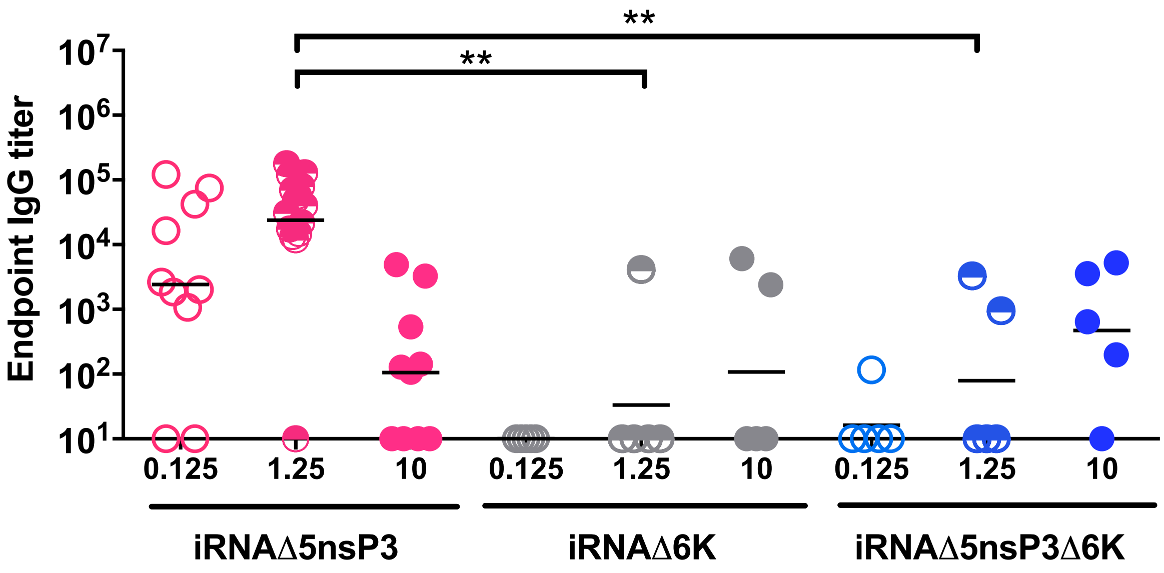

Supplementary Figure 3. Immunogenicity of three iRNA vaccine candidates. C57BL/6J mice were immunized once with indicated doses of candidate vaccines. Total antigen-specific IgG titers were determined by ELISA three weeks post immunization with a cut-off value set at 0.1. The line indicates the geometric mean of each group (n=5-15 animals per group). A Kruskal-Wallis test followed by Dunn's posttest was used to compare the responses of the different groups. Two asterisks (**) indicate statistical differences of $p < 0.01$
